# Cerebellar Structure and Function in Autism Spectrum Disorder

**DOI:** 10.20900/jpbs.20220003

**Published:** 2022-06-27

**Authors:** Bess F. Bloomer, Jaime J. Morales, Amanda R. Bolbecker, Dae-Jin Kim, William P. Hetrick

**Affiliations:** 1 Department of Psychological and Brain Sciences, Indiana University, 1101 E. 10^th^ St., Bloomington, IN 47405, USA; 2 Program in Neuroscience, Indiana University, Indiana University, 1101 E. 10^th^ St., Bloomington, IN 47405, USA; 3 Department of Psychiatry, Indiana University School of Medicine, Indianapolis, IN 46202, USA

**Keywords:** autism spectrum disorder, cerebellum, development, cognition

## Abstract

Autism spectrum disorder (ASD) is a heterogeneous neurodevelopmental condition characterized by early-onset repetitive behaviors, restricted interests, sensory and motor difficulties, and impaired social interactions. Converging evidence from neuroimaging, lesion and postmortem studies, and rodent models suggests cerebellar involvement in ASD and points to promising targets for therapeutic interventions for the disorder. This review elucidates understanding of cerebellar mechanisms in ASD by integrating and contextualizing recent structural and functional cerebellar research.

## INTRODUCTION: CEREBELLUM, CEREBELLAR DEVELOPMENT, AND ASD

Autism spectrum disorder (ASD) is a heterogeneous neurodevelopmental condition characterized by early-onset repetitive behaviors, restricted interests, sensory and motor difficulties, and impaired social interactions [1]. There is increasing interest in understanding cerebellar contributions to ASD, given growing evidence of structural and functional anomalies in ASD [2,3]. For example, human postmortem studies report Purkinje cell degeneration and loss [4–7] and GABA and reelin abnormalities [8]. Moreover, these cerebellar structural abnormalities have been linked to ASD symptom emergence. For example, core ASD symptoms such as decreased exploratory behavior and repetitive, stereotyped behavior have been significantly correlated with cerebellar vermis volume reduction [9]. In addition, lobular cerebellar gray matter volume is inversely correlated with ASD symptom severity, specifically social interaction, communication, and repetitive behaviors [10]. Riva and colleagues [11] found that children with ASD had reduced cerebellar grey matter volume in Crus II and vermis, which was associated with reduced communication and interaction, suggesting cerebellar involvement in social interaction deficits seen in ASD. A recent study by Kelly et al. [12] used optogenetic techniques in a mouse model of ASD to demonstrate reduced functional connectivity between the right cerebellar Crus I and medial prefrontal cortex (PFC), a finding they corroborated with fMRI analyses of brain connectivity in individuals with ASD. This finding suggests that abnormal development of cerebellar-cortical circuits likely contribute to social cognitive processes that are often deficient in ASD. More thorough examination into early development of the cerebellum may provide further insight into the processes by which these circuits become dysfunctional.

The cerebellum is a complex and late-maturing neural region that is vulnerable to early developmental insults that can profoundly disturb intracerebellar development [13]. Consequently, cerebellar developmental disturbances are believed to impair proper cerebellar-cortical circuit formation, which are robust findings in ASD [14–16].

Inhibitory Purkinje cells and excitatory granule cells are integral to proper pre- and post-natal development. Disruption of either of these two cell systems can profoundly disrupt cerebellar development [17,18]. In recent years, theories have emerged suggesting that the etiology and development of ASD is partially caused by widespread mild but cumulative disruptions to the Purkinje cell system [19,20].

One significant way cerebellar development may be impaired is through disturbances in precisely timed and reciprocal interactions of granule and Purkinje cell, which are critical to later prenatal stages of cerebellar brain development [21]. Granule cell neurogenesis is promoted by the earlier-born Purkinje cells [18]. Additionally, granule cells are key components to the functioning of the cerebellum as they make up most of the synaptic input onto Purkinje cell dendrites and are fundamental to the cerebellar cortex’s lamination process during development [22]. Thus, early genetic, mechanical, or environmental disruptions to this developmental cascade can be amplified, causing damage not only within cerebellum, but also disrupting the development of critical cerebellar-cortical circuits.

The above findings collectively suggest cerebellar involvement in ASD and point to promising targets for therapeutic interventions. The aim of the current review is to explicate understanding of cerebellar mechanisms in ASD by integrating and contextualizing recent structural and functional cerebellar research in ASD using evidence from studies in neuroimaging, lesions, postmortem, and murine models. There is specific emphasis on the mechanisms by which the cerebellum contributes to social-cognitive processes within ASD. For other recent reviews on the topic of the cerebellum and ASD, see Mapelli et al. [23].

## EVIDENCE FOR CEREBELLAR INVOLVEMENT IN SOCIAL COGNITION

The internal working models within the cerebellum allow for feedforward predictive motor control contributing to coordinated movement [24,25]. During early development and throughout the lifespan, the cerebellum helps to make the functioning of the rest of the cerebral cortex more efficient through its modulation of other significant cortical areas via the feedforward mechanisms it uses to update one’s internal models of the world [26]. Just as the cerebellum plays a crucial role in motor control, balance, and coordination via the formation of internal models, it also uses similar adaptive feedforward mechanisms to modulate non-motor psychological processes [26–28]. Increasing evidence supports the cerebellum’s importance beyond basic motor control to include important aspects of non-motor cognition. When cognitive processes are more effortful, there is a higher degree of engagement from the cerebellum, leading to the suggestion that cerebellum moderates higher order cognition by facilitating the efficiency of other cortical regions by ways of sending adaptive feedback to the cerebral cortex [29]. Further, anatomical tracing has shown extensive communication between most of the cerebrum, including areas which are not typically implicated in motor processes, and the cerebellum [30,31]. This framework for understanding the cerebellum underpins the theory that the cerebellum modulates cognitive processes by updating existing knowledge and sending adaptive feedback to other cortical regions [26,27]. For a more comprehensive review of the cerebellum’s role in cognitive states, see Schmahmann [32].

Considering that core symptoms of ASD often involve impairments in social interaction and interpersonal communication, social cognitive processes such as mentalizing abilities and social prediction are important areas of study [1,33]. Growing evidence has implicated the cerebellum’s contribution to social processes, such as thinking of other’s cognitive or emotional states. For example, Van Overwalle and colleagues [29] conducted a meta-analysis of over 350 fMRI studies which revealed that abstraction processes in social cognition (e.g., thinking about oneself in the future and recalling autobiographical past) activated various regions within the cerebellum in non-clinical populations. Given the cerebellum appears to be integral in social cognitive processes also impaired in ASD, recent theories of adaptive social prediction have begun to ascribe cerebellar contributions to the anticipation of a social partner’s thoughts and intentions or to make inferences of other’s mental states [29,34–36].

Social deficits in autistic individuals may arise due to difficulty in using past information to flexibly adjust social behavior and adapt to changing social situations [37], which can be interpreted as a failure to update internal models of one’s social world. Fundamental social cognitive processes used in many day-to-day social and communicative functions seem to be mediated by Crus I and II–PFC circuit connectivity, and this network has been found to be particularly affected in ASD as the impairment of this circuit seems to contribute to core symptoms of the disorder [35,38,39]. It is important to note that findings in ASD consistently point toward a reduction of volume in posterior Crus I/II lobules [34], areas which have been implicated in multiple social cognitive processes [29,40,41].

Important processes for successful social interactions involve theory of mind (understanding how another person is thinking and/or feeling based on one’s understanding of the self and other external individuals), body reading (understanding bodily gestures to help in inference of social interactions), and emotion recognition (construing another’s emotional expression) which are all commonly affected in ASD. In ASD, reduced cerebellar activation has been shown during mirroring actions or mental states of others [42]. In non-autistic individuals, theory of mind/mentalizing tasks reliably engage the Crus I and Crus II lobules, with these cerebellar regions also showing strong connectivity with key regions of mentalizing such as the medial prefrontal cortex and the tempo-parietal junction [9,43].

Exploration of the perception of biological motion of the human body in ASD has revealed decreased connectivity between several areas within the cerebrum and posterior cerebellum during body reading—specifically, negative associations between level of social interaction impairment and Crus I/II activation have been demonstrated in this population [44]. In non-clinical populations, activations of Crus I, lobule VIIB, lobule VI, and Crus II seem to be present during body reading tasks [29,45]. Sokolov and colleagues [45] further found functional connectivity between the superior temporal sulcus and cerebellum, pointing to pathways that help facilitate communication between the cerebrum and cerebellum during social cognitive processes.

Autistic individuals often present with profound facial emotion recognition deficits [46], and how the cerebellum contributes to emotion recognition processes in ASD is not understood. However, numerous studies point to cerebellar activation during facial emotion recognition in non-clinical populations [41,47], primarily in posterior cerebellar regions such as Crus I/II and lobule VI, which suggest that these cerebellar regions should be a focus in future studies of emotional recognition in ASD.

These recent empirical findings not only reinforce the notion that the cerebellum is an integral brain structure involved in creating feedforward models of our environment, but also provide evidence that it plays a distinct role in higher order social cognitive processes that are impaired in ASD.

## CEREBELLAR INJURY ASSOCIATED WITH ASD

Lesion studies have also contributed to the present understanding of ASD due to the sometimes drastic neural, behavioral, and clinical consequences following early damage to the cerebellum. Cerebellar injury is a frequent finding in very preterm infants (<32 weeks), and up to 40% of all infants who have cerebellar lesions or hemorrhages are diagnosed with ASD [21,48]. During weeks 20–40 of gestation, the cerebellum is the most rapidly developing brain structure [49,50]. Therefore, cerebellar development is disrupted in preterm infants during a critical neurodevelopmental window [21].

Postnatal cerebellar damage can also have adverse developmental consequences. For example, Boswinkel et al. [19] found that children with cerebellar hemorrhage have altered developmental trajectories, and the severity of abnormal neurodevelopmental outcome is associated with the severity of cerebellar hemorrhage. One example of cerebellar injury leading to ASD-like symptoms is that of posterior fossa syndrome. This syndrome includes symptoms that mirror ASD such as language deficits, difficulties with spatial cognition, disinhibited or inappropriate behavior, and problems with modulating affect [14,51]. Many children who have medulloblastoma cerebellar tumor resection surgery go on to experience posterior fossa syndrome, with the extent of the resection related to the later development of this syndrome [52]. Similarly, positive correlations were found between vermal abnormalities and ASD symptomatology in large studies of children with cerebellar malformations on cerebellar malformations, supporting a link between ASD and cerebellar pathology [53].

Given the considerable overlap of ASD diagnosis and cerebellar lesions, it is also important to note that patients with cerebellar injury and/or lesions performed worse on mentalizing tasks, providing further evidence of the cerebellum’s role in the social-cognitive process of interpretation of other’s mental states [54]. In addition, children with cerebellar lesions have shown difficulty in using other’s actions to predict their ultimate social outcomes [55]. Because cerebellar lesions are associated with lower performance in social processes commonly impacted by a diagnosis of autism, injury to this important neural region should not be understated.

Taken together, evidence indicates that the cerebellum follows a complex developmental trajectory which is highly sensitive to insult such as premature birth and early cerebellar injury and that autistic-like clinical symptoms are associated with cerebellar injury.

## MOUSE MODELS SUPPORT CEREBELLAR INVOLVEMENT IN ASD

Supporting the putative role of the cerebellum in ASD, mouse models of autism frequently show cerebellar abnormalities [56]. Mouse models of ASD which involve specific deletions or mutations of candidate genes shown to be influential in the development of the disorder, such as CADPS2 and GABRB3. For a more detailed review on animal models and ASD, see Mapelli et al. [23].

For example, rare variants of the gene CADPS2 have been found to be associated with ASD [57]. Simiarly, CADPS2-KO (knockout) mice exhibit autistic-like behaviors such as impaired sociability and higher anxiety with novelty [58]. GABRB3 has also been identified as a gene of interest, as GABRB3 gene expression has been found to be reduced within the cerebellum of autistic individuals [59]. GABRB3-KO mice show both cerebellar structural abnormalities of cerebellar hypoplasia, but also hyperactivity, poor motor skills, and decreased social behavior consistent with what is often found in ASD [60]. One well studied mouse model that links cerebellar abnormalities to ASD-like behaviors is tuberous sclerosis complex (TSC), a genetic disorder that results from mutation of the TSC1 or TSC2 gene and is associated with high rates of comorbid ASD in humans. TSC1 and TSC2 mutant mice display reduced interest in exploratory social behavior [61], increased rates of repetitive self-grooming, and increased cognitive rigidity as demonstrated by impaired learning of a new escape platform [62]. Kelly et al. [12] demonstrated that TSC1 mutant mice showed hyperactivity of the medial prefrontal cortex, and chemo-genetic inhibition of mPFC activity reduced repetitive grooming in the mice as well as improving flexibility in solving a water maze task, which mirror common behavioral patterns found in ASD, suggesting that these mPFC-cerebellar circuits mediate these ASD-like behaviors.

In mice, chemo-genetically mediated inhibition of Purkinje cells results in ASD-related social and repetitive behaviors, and inhibition of interneurons that directly influence firing activity of Purkinje cells in the right Crus I. Interestingly, ASD-like social and repetitive behaviors are influenced by this inhibition but not motor and gait behavior, suggesting an independent and unique contribution to social and repetitive behaviors by the cerebellum beyond motor dysfunction [63]. By manipulating neural activity in the mouse cerebellum using reversible chemo-genetic perturbation of molecular layer interneurons, Badura et al. [64] demonstrated that Crus I plays a role in the development of reversal learning, novelty-seeking, and most prominently, social preference. Increasing rodent model evidence clearly suggests that these cerebellar mechanisms may be disturbed in ASD, and these disruptions may lead to downstream consequences resulting in ASD-like symptom expression and behavior. Likewise, these rodent findings support a key role for cerebellum for the emergence of normal social-cognitive development.

## CONCLUSION

In summary, cerebellar structural and functional abnormalities are commonly reported in ASD, and evidence of cerebellar contributions to social-cognitive deficits in rodent models of ASD convincingly converge with these findings. It is important to use the collective findings gathered from recent neuroimaging studies, mouse models, meta-analyses, lesion studies, and neuropathological studies to add to the field’s growing knowledge of the dysfunction of the cerebellum in ASD so that more efficacious and mechanism-based treatments for this heterogeneous condition can be identified.

## Figures and Tables

**Figure 1.**
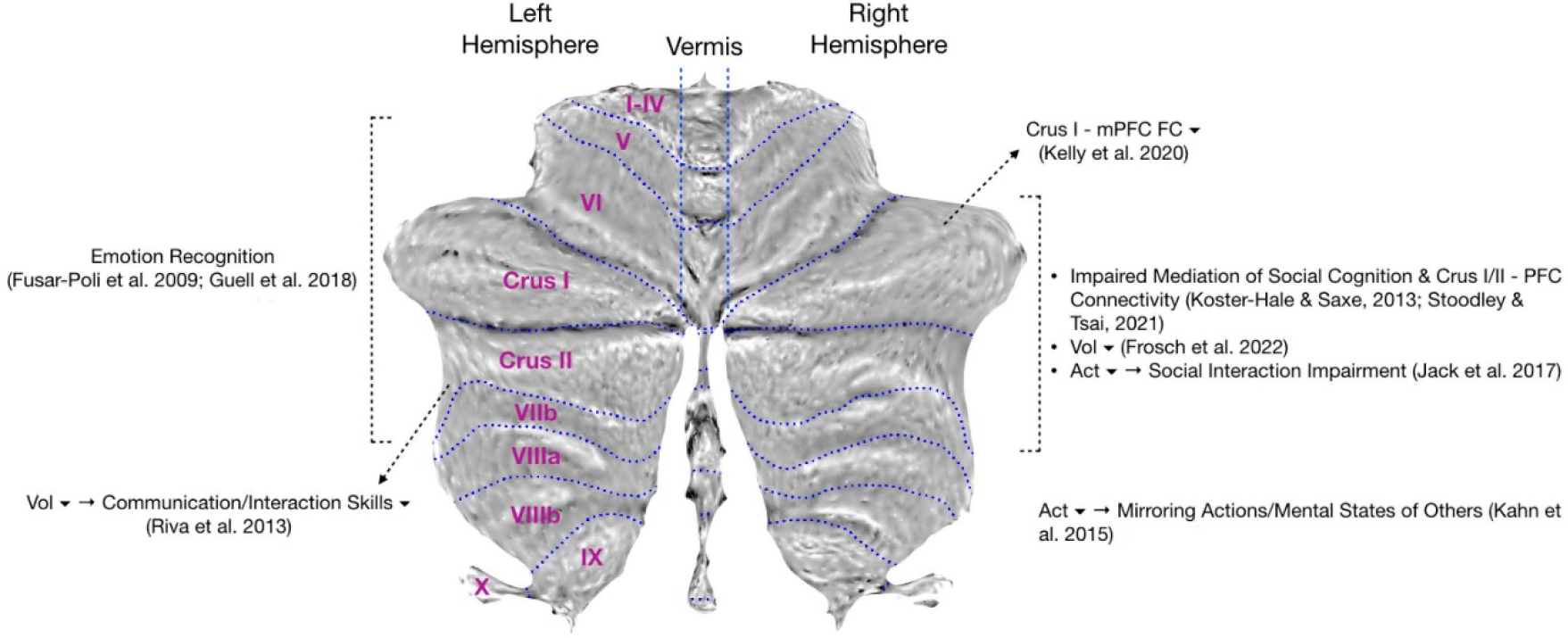
A visual summary of ASD-specific cerebellar differences.

## Data Availability

No data were generated from this study.

## References

[1] American Psychiatric Association. Diagnostic and statistical manual of mental disorders. 5th ed. Washington, (D.C., US) American Psychiatric Association; 2013.

[2] Kemper TL, Bauman M. Neuropathology of Infantile Autism: J Neuropathol Exp Neurol. 1998;57(7):645–52. 9690668 10.1097/00005072-199807000-00001

[3] Stoodley CJ, Limperopoulos C. Structure–function relationships in the developing cerebellum: Evidence from early-life cerebellar injury and neurodevelopmental disorders. Semin Fetal Neonatal Med. 2016;21(5):356–64. 27184461 10.1016/j.siny.2016.04.010 PMC5282860

[4] Bailey A A clinicopathological study of autism. Brain. 1998;121(5):889–905. 9619192 10.1093/brain/121.5.889

[5] Bauman ML, Kemper TL. Neuroanatomic observations of the brain in autism: A review and future directions. Int J Dev Neurosci. 2005;23(2–3):183–7. 15749244 10.1016/j.ijdevneu.2004.09.006

[6] Becker EBE, Stoodley CJ. Autism Spectrum Disorder and the Cerebellum. Int Rev Neurobiol. 2013;113:1–34. 24290381 10.1016/B978-0-12-418700-9.00001-0

[7] Whitney ER, Kemper TL, Rosene DL, Bauman ML, Blatt GJ. Density of cerebellar basket and stellate cells in autism: Evidence for a late developmental loss of Purkinje cells. J Neurosci Res. 2009;87(10):2245–54. 19301429 10.1002/jnr.22056 PMC2760265

[8] Fatemi SH, Aldinger KA, Ashwood P, Bauman ML, Blaha CD, Blatt GJ, Consensus paper: pathological role of the cerebellum in autism. Cerebellum. 2012;11(3), 777–807. 10.1007/s12311-012-0355-9 22370873 PMC3677555

[9] Pierce K, Courchesne E. Evidence for a cerebellar role in reduced exploration and stereotyped behavior in autism. Biol Psychiatry. 2001;49(8):655–64. 11313033 10.1016/s0006-3223(00)01008-8

[10] D’Mello AM, Crocetti D, Mostofsky SH, Stoodley CJ. Cerebellar gray matter and lobular volumes correlate with core autism symptoms. NeuroImage. 2015;7:631–9. 25844317 10.1016/j.nicl.2015.02.007 PMC4375648

[11] Riva D, Annunziata S, Contarino V, Erbetta A, Aquino D, Bulgheroni S. Gray matter reduction in the vermis and CRUS-II is associated with social and interaction deficits in low-functioning children with autistic spectrum disorders: A VBM-DARTEL study. Cerebellum. 2013;12(5):676–85. 23572290 10.1007/s12311-013-0469-8

[12] Kelly E, Meng F, Fujita H, Morgado F, Kazemi Y, Rice LC, Regulation of autism-relevant behaviors by cerebellar–prefrontal cortical circuits. Nat Neurosci. 2020;23(9):1102–10. 32661395 10.1038/s41593-020-0665-z PMC7483861

[13] Sathyanesan A, Zhou J, Scafidi J, Heck DH, Sillitoe RV, Gallo V. Emerging connections between cerebellar development, behaviour and complex brain disorders. Nat Rev. Neurosci. 2019;20(5):298–313. 30923348 10.1038/s41583-019-0152-2 PMC7236620

[14] Gill JS, Sillitoe RV. Functional Outcomes of Cerebellar Malformations. Front Cell Neurosci. 2019;13:441. 31636540 10.3389/fncel.2019.00441 PMC6787289

[15] Stoodley CJ. Distinct regions of the cerebellum show gray matter decreases in autism, ADHD, and developmental dyslexia. Front Syst Neurosci. 2014;8:92. 24904314 10.3389/fnsys.2014.00092 PMC4033133

[16] Stoodley CJ. The Cerebellum and Neurodevelopmental Disorders. Cerebellum. 2016;15(1):34–7. 26298473 10.1007/s12311-015-0715-3 PMC4811332

[17] van der Heijden ME, Lackey EP, Işleyen FS, Brown AM, Perez R, Lin T, Maturation of Purkinje cell firing properties relies on granule cell neurogenesis. Elife. 2021 Sep 20;10:e68045. 34542409 10.7554/eLife.68045 PMC8452305

[18] van der Heijden ME, Sillitoe RV. Interactions Between Purkinje Cells and Granule Cells Coordinate the Development of Functional Cerebellar Circuits. In Memoriam: Masao Ito—A Visionary Neuroscientist with a Passion for the Cerebellum. Neuroscience. 2021;462:4–21. 32554107 10.1016/j.neuroscience.2020.06.010 PMC7736359

[19] Boswinkel V, Steggerda SJ, Fumagalli M, Parodi A, Ramenghi LA, Groenendaal F, The CHOPIn Study: A Multicenter Study on Cerebellar Hemorrhage and Outcome in Preterm Infants. Cerebellum. 2019;18(6):989–98. 10.1007/s12311-019-01053-1 31250213

[20] Herzmann CS, Snyder AZ, Kenley JK, Rogers CE, Shimony JS, Smyser CD. Cerebellar Functional Connectivity in Term- and Very Preterm-Born Infants. Cereb Cortex. 2019;29(3): 1174–84. 29420701 10.1093/cercor/bhy023 PMC6373668

[21] van der Heijden ME, Gill JS, Sillitoe RV. Abnormal Cerebellar Development in Autism Spectrum Disorders. Dev Neurosci. 2021;43(3–4):181–90. 33823515 10.1159/000515189 PMC8440334

[22] Consalez GG, Goldowitz D, Casoni F, Hawkes R. Origins, Development, and Compartmentation of the Granule Cells of the Cerebellum. Front Neural Circuits. 2021;14:611841. 33519389 10.3389/fncir.2020.611841 PMC7843939

[23] Mapelli L, Soda T, D’Angelo E, Prestori F. The Cerebellar Involvement in Autism Spectrum Disorders: From the Social Brain to Mouse Models. Int J Mol Sci. 2022;23(7):3894. 35409253 10.3390/ijms23073894 PMC8998980

[24] Bhanpuri NH, Okamura AM, Bastian AJ. Predictive modeling by the cerebellum improves proprioception. J Neurosci. 2013;33(36):14301–14306. 24005283 10.1523/JNEUROSCI.0784-13.2013 PMC3761044

[25] Ito M Control of mental activities by internal models in the cerebellum. Nat Rev Neurosci. 2008;9(4):304–13. 18319727 10.1038/nrn2332

[26] Andreasen NC, Pierson R. The role of the cerebellum in schizophrenia. Biol Psychiatry. 2008;64(2):81–8. 18395701 10.1016/j.biopsych.2008.01.003 PMC3175494

[27] Bower JM. Chapter 27 Is the cerebellum sensory for motor’s sake, or motor for sensory’s sake: The view from the whiskers of a rat? Prog Brain Res. 1997;114:463–96. 9193161 10.1016/s0079-6123(08)63381-6

[28] Wang SSH, Kloth AD, Badura A. The cerebellum, sensitive periods, and autism. Neuron. 2014;83(3):518–32. 25102558 10.1016/j.neuron.2014.07.016 PMC4135479

[29] Van Overwalle F, Baetens K, Mariën P, Vandekerckhove M. Social cognition and the cerebellum: A meta-analysis of over 350 fMRI studies. NeuroImage. 2014;86:554–72. 24076206 10.1016/j.neuroimage.2013.09.033

[30] Strick PL, Dum RP, Fiez JA. Cerebellum and nonmotor function. Ann Rev Neurosci. 2009;32, 413–4. 19555291 10.1146/annurev.neuro.31.060407.125606

[31] Krienen FM, Buckner RL. Segregated fronto-cerebellar circuits revealed by intrinsic functional connectivity. Cereb Cortex. 2009;19(10):2485–97. 19592571 10.1093/cercor/bhp135 PMC2742600

[32] Schmahmann JD. The cerebellum and cognition. Neurosci Lett. 2019;688:62–75. 29997061 10.1016/j.neulet.2018.07.005

[33] Farrar MJ, Seung HK, Lee H. Language and False-Belief Task Performance in Children With Autism Spectrum Disorder. J Speech Lang Hear Res. 2017 Jul 12;60(7):1999–2013. 28666276 10.1044/2017_JSLHR-L-15-0422

[34] Frosch IR, Mittal VA, & D’Mello AM. Cerebellar Contributions to Social Cognition in ASD: A Predictive Processing Framework. Front Integr Neurosci. 2022;16:810425. 35153691 10.3389/fnint.2022.810425 PMC8832100

[35] Koster-Hale J, Saxe R. Theory of mind: a neural prediction problem. Neuron. 2013;79(5):836–48. 24012000 10.1016/j.neuron.2013.08.020 PMC4041537

[36] Kozareva V, Martin C, Osorno T, Rudolph S, Guo C, Vanderburg C, A transcriptomic atlas of mouse cerebellar cortex comprehensively defines cell types. Nature. 2021);598(7879):214–9. 34616064 10.1038/s41586-021-03220-z PMC8494635

[37] Cannon J, O’Brien AM, Bungert L, Sinha P. Prediction in Autism Spectrum Disorder: A Systematic Review of Empirical Evidence. Autism Res. 2021;14(4):604–30. 33570249 10.1002/aur.2482 PMC8043993

[38] Stoodley CJ, Tsai PT. Adaptive Prediction for Social Contexts: The Cerebellar Contribution to Typical and Atypical Social Behaviors. Ann Rev Neurosci. 2021;44(1):475–93. 34236892 10.1146/annurev-neuro-100120-092143 PMC9037460

[39] Rogers TD, McKimm E, Dickson PE, Goldowitz D, Blaha CD, Mittleman G. Is autism a disease of the cerebellum? An integration of clinical and pre-clinical research. Front Syst Neurosci. 2013;7:15. 23717269 10.3389/fnsys.2013.00015 PMC3650713

[40] Ernst TM, Brol AE, Gratz M, Ritter C, Bingel U, Schlamann M, The cerebellum is involved in processing of predictions and prediction errors in a fear conditioning paradigm. ELife. 2019;8:e46831. 31464686 10.7554/eLife.46831 PMC6715348

[41] Guell X, Gabrieli J, Schmahmann JD. Triple representation of language, working memory, social and emotion processing in the cerebellum: convergent evidence from task and seed-based resting-state fMRI analyses in a single large cohort. NeuroImage. 2018;172:437–49. 29408539 10.1016/j.neuroimage.2018.01.082 PMC5910233

[42] Khan AJ, Nair A, Keown CL, Datko MC, Lincoln AJ, Müller R-A. Cerebro-cerebellar Resting-State Functional Connectivity in Children and Adolescents with Autism Spectrum Disorder. Biol Psychiatry. 2015;78(9:625–34. 25959247 10.1016/j.biopsych.2015.03.024 PMC5708535

[43] Van Overwalle F, Ma Q, Heleven E. The posterior crus II cerebellum is specialized for social mentalizing and emotional self-experiences: A meta-analysis. Soc Cogn Affect Neurosci. 2020;15(9):905–28. 32888303 10.1093/scan/nsaa124 PMC7851889

[44] Jack A, Keifer CM, Pelphrey KA. Cerebellar contributions to biological motion perception in autism and typical development. Hum Brain Mapp. 2017;38(4):1914–32. 28150911 10.1002/hbm.23493 PMC5342927

[45] Sokolov AA, Erb M, Gharabaghi A, Grodd W, Tatagiba MS, Pavlova MA. Biological motion processing: the left cerebellum communicates with the right superior temporal sulcus. NeuroImage. 2012;59(3):2824–30. 22019860 10.1016/j.neuroimage.2011.08.039

[46] Loth E, Garrido L, Ahmad J, Watson E, Duff A, Duchaine B. Facial expression recognition as a candidate marker for autism spectrum disorder: How frequent and severe are deficits? Mol Autism. 2018 Jan 30;9:7. 29423133 10.1186/s13229-018-0187-7 PMC5791186

[47] Fusar-Poli P, Placentino A, Carletti F, Landi P, Allen P, Surguladze S, Functional atlas of emotional faces processing: a voxel-based meta-analysis of 105 functional magnetic resonance imaging studies. J Psychiatry Neurosci. 2009;34(6):418–32. 19949718 PMC2783433

[48] Steggerda SJ, Leijser LM, Wiggers-de Bruïne FT, van der Grond J, Walther FJ, van Wezel-Meijler G. Cerebellar Injury in Preterm Infants: Incidence and Findings on US and MR Images. Radiology. 2009;252(1):190–9. 19420320 10.1148/radiol.2521081525

[49] Clouchoux C, Kudelski D, Gholipour A, Warfield SK, Viseur S, Bouyssi-Kobar M, Quantitative in vivo MRI measurement of cortical development in the fetus. Brain Struct Funct. 2012 Jan;217(1):127–39. 21562906 10.1007/s00429-011-0325-x

[50] Limperopoulos C, Soul JS, Gauvreau K, Huppi PS, Warfield SK, Bassan H, Late gestation cerebellar growth is rapid and impeded by premature birth. Pediatrics. 2005;115(3):688–95. 15741373 10.1542/peds.2004-1169

[51] Levisohn L, Cronin-Golomb A, Schmahmann JD. Neuropsychological consequences of cerebellar tumour resection in children: Cerebellar cognitive affective syndrome in a paediatric population. Brain. 2000;123(5):1041–50. 10775548 10.1093/brain/123.5.1041

[52] Korah MP, Esiashvili N, Mazewski CM, Hudgins RJ, Tighiouart M, Janss AJ, Incidence, risks, and sequelae of posterior fossa syndrome in pediatric medulloblastoma. Int J Radiat Oncol Biol Phys. 2010;77(1):106–12. 19695790 10.1016/j.ijrobp.2009.04.058

[53] Bolduc ME, Du Plessis AJ, Sullivan N, Guizard N, Zhang X, Robertson RL, Regional cerebellar volumes predict functional outcome in children with cerebellar malformations. Cerebellum. 2012;11(2):531–542 21901523 10.1007/s12311-011-0312-z

[54] Van Overwalle F, De Coninck S, Heleven E, Perrotta G, Taib NOB, Manto M, The role of the cerebellum in reconstructing social action sequences: A pilot study. Soc Cogn Affect Neurosci. 2019;14(5):549–58. 31037308 10.1093/scan/nsz032 PMC6545532

[55] Clausi S, Olivito G, Siciliano L, Lupo M, Laghi F, Baiocco R, The cerebellum is linked to theory of mind alterations in autism. A direct clinical and MRI comparison between individuals with autism and cerebellar neurodegenerative pathologies. Autism Res. 2021;14(11):2300–13. 34374492 10.1002/aur.2593 PMC9291804

[56] Kloth AD, Badura A, Li A, Cherskov A, Connolly SG, Giovannucci A, Cerebellar associative sensory learning defects in five mouse autism models. eLife. 2015;4:e06085. 26158416 10.7554/eLife.06085 PMC4512177

[57] Bonora E, Graziano C, Minopoli F, Bacchelli E, Magini P, Diquigiovanni C, Maternally inherited genetic variants of CADPS2 are present in autism spectrum disorders and intellectual disability patients. EMBO Mol Med. 2014;6(6):795–809. 24737869 10.1002/emmm.201303235 PMC4203356

[58] Sadakata T, Kakegawa W, Mizoguchi A, Washida M, Katoh-Semba R, Shutoh F, Impaired cerebellar development and function in mice lacking CAPS2, a protein involved in neurotrophin release. J Neurosci. 2007;27(10):2472–82. 17344385 10.1523/JNEUROSCI.2279-06.2007 PMC6672497

[59] Homanics GE, DeLorey TM, Firestone LL, Quinlan JJ, Handforth A, Harrison NL, Mice devoid of gamma-aminobutyrate type A receptor beta3 subunit have epilepsy, cleft palate, and hypersensitive behavior. Proc Nat Acad Sci U S A. 1997;94(8):4143–48. 10.1073/pnas.94.8.4143 PMC20582 9108119

[60] DeLorey TM, Sahbaie P, Hashemi E, Homanics GE, Clark JD. Gabrb3 gene deficient mice exhibit impaired social and exploratory behaviors, deficits in non-selective attention and hypoplasia of cerebellar vermal lobules: a potential model of autism spectrum disorder. Behav Brain Res. 2008;187(2):207–20. 17983671 10.1016/j.bbr.2007.09.009 PMC2684890

[61] Young DM, Schenk AK, Yang S-B, Jan YN, Jan LY. Altered ultrasonic vocalizations in a tuberous sclerosis mouse model of autism. Proc Nat Acad Sci U S A. 2010);107(24):11074–9. 10.1073/pnas.1005620107 PMC2890736 20534473

[62] Tsai PT, Hull C, Chu Y, Greene-Colozzi E, Sadowski AR, Leech JM, Autistic-like behaviour and cerebellar dysfunction in Purkinje cell Tsc1 mutant mice. Nature. 2012;488(7413):647–51. 22763451 10.1038/nature11310 PMC3615424

[63] Stoodley CJ, D’Mello AM, Ellegood J, Jakkamsetti V, Liu P, Nebel MB, Altered cerebellar connectivity in autism and cerebellar-mediated rescue of autism-related behaviors in mice. Nat Neurosci. 2017;20(12):1744–51. 29184200 10.1038/s41593-017-0004-1 PMC5867894

[64] Badura A, Verpeut JL, Metzger JW, Pereira TD, Pisano TJ, Deverett B, Normal cognitive and social development require posterior cerebellar activity. ELife. 2018;7:e36401. 30226467 10.7554/eLife.36401 PMC6195348

